# Evolution of sexual asymmetry

**DOI:** 10.1186/1471-2148-4-34

**Published:** 2004-09-21

**Authors:** Tamás L Czárán, Rolf F Hoekstra

**Affiliations:** 1 Theoretical Biology and Ecology Research Group of the Hungarian Academy of Sciences and Eötvös University, H-1117 Budapest, Pázmány Péter sétány 1/c, Hungary; 2 Laboratory of Genetics, Department of Plant Sciences, Wageningen University, Arboretumlaan 4, 6703 BD Wageningen, The Netherlands

## Abstract

**Background:**

The clear dominance of two-gender sex in recent species is a notorious puzzle of evolutionary theory. It has at least two layers: besides the most fundamental and challenging question why sex exists at all, the other part of the problem is equally perplexing but much less studied. Why do most sexual organisms use a binary mating system? Even if sex confers an evolutionary advantage (through whatever genetic mechanism), why does it manifest that advantage in two, and exactly two, genders (or mating types)? Why not just one, and why not more than two?

**Results:**

Assuming that sex carries an inherent fitness advantage over pure clonal multiplication, we attempt to give a feasible solution to the problem of the evolution of dimorphic sexual asymmetry as opposed to monomorphic symmetry by using a spatial (cellular automaton) model and its non-spatial (mean-field) approximation. Based on a comparison of the spatial model to the mean-field approximation we suggest that spatial population structure must have played a significant role in the evolution of mating types, due to the largely clonal (self-aggregated) spatial distribution of gamete types, which is plausible in aquatic habitats for physical reasons, and appears to facilitate the evolution of a binary mating system.

**Conclusions:**

Under broad ecological and genetic conditions the cellular automaton predicts selective removal from the population of supposedly primitive gametes that are able to mate with their own type, whereas the non-spatial model admits coexistence of the primitive type and the mating types. Thus we offer a basically ecological solution to a theoretical problem that earlier models based on random gamete encounters had failed to resolve.

## Background

One of the most general rules in biology seems to be that sex involves the fusion of gametes (sometimes of other specialised structures) of different type. In most taxa this sexual asymmetry is reflected in the male / female distinction between mating partners and/or between mating sex cells. This paper aims to help understand why sex is asymmetric.

The primary difference between male and female is anisogamy, the differential size and mobility of gametes. Anisogamy is thought to have evolved from a more primitive condition of isogamy (for reviews see [1]; [2] see also [3]).

In isogamous species without apparent male-female differentiation, like unicellular green algae (e.g. *Chlamydomonas*) and fungi (e.g. yeast), the asymmetry in sexual fusion and subsequent development are regulated by a binary mating type system. Mating is only possible between cells of different mating type. Molecular analysis has revealed a remarkable and complex genetic mating type structure [4,5]. The two mating types in a species consist of so-called idiomorphs [6], non-homologous complexes of closely linked genes that occupy homologous positions at the same chromosomal locus. They behave as alleles in being mutually exclusive in meiotic segregation. A similar binary mating type system exists in many filamentous ascomycetous fungi [7], which however often also exhibit male / female differentiation. Only matings between individuals of different mating type are allowed. Thus in mycelia that can function both as male and as female self-mating is prevented. Mating in such species is heterothallic, that is, always between different individuals. However, many ascomycetes are homothallic, i.e. can complete the sexual cycle in a single individual. Homothallic species may lack mating types, such as *Aspergillus nidulans*, or may consist of individuals that are heterokaryotic for mating type (carry nuclei of both mating types) such as *Podospora anserina. *In the latter case sexual fusion is between different mating types at the nuclear level, but can occur within a single individual mycelium.

In basidiomycetous fungi, morphological sexual differentiation is absent, but mating is regulated by complex mating systems, generating in some cases large numbers of different mating types. Also here, the mating type genes control sexual fusion and post-fusion development [8]. Again, mating cannot occur between individuals of the same mating type.

In other taxa still other genetic systems exist that control sexual fusion, sometimes in addition to the male-female difference. In monoecious higher plants often self-incompatibility systems occur that effectively exclude self-mating [9,10]. Among ciliates, several variations on the theme of mating type differentiation exist, which are not further detailed here.

All these different mating systems have one characteristic in common: mating is always asymmetric. When gender differences exist, mating involves the fusion of a male and a female cell; this may occur when the male and female functions are in different individuals, or when a single individual possesses both male and female functions. When gender differentiation is absent, mating type systems guarantee that sexual fusions are between different types. However, the absence of both gender and mating type differentiation has never been observed. This would imply symmetric sexual fusion: a species in which every sex cell could potentially fuse with any other sex cell. Because gender differences starting with anisogamy most likely evolved from pre-existing isogamy, we should consider the evolution of mating types in an isogamous species to understand why sex is asymmetric.

Functional explanations of the evolution of a binary mating type system have been explored in theoretical models by [11-13] and [14]. These models differ in their biological assumptions. According to [12] and [13], mating types have evolved to suppress harmful conflicts between cytoplasmic elements, while [11] suggests that mating type loci have evolved in response to polymorphisms for genes involved in gamete recognition. It is still not possible to conclusively decide between the alternative biological scenario's [15]. However, all models envisage as a starting point an initially undifferentiated population in which every gamete can mate with any other gamete, and derive conditions for the evolution of two mating types that exclusively mate with each other and have lost the ability to mate with their own type. A general conclusion emerging from the models is that mating types may invade the initially undifferentiated population under fairly broad conditions, but that the removal of the undifferentiated type requires very strong selective forces. It is this latter aspect which in our view still forms a problem, because it is difficult to see why the original type should be so disadvantageous compared to the differentiated mating types. The mentioned models assume a homogeneous population in which random encounters lead to mating. However, this assumption is likely to be very unrealistic if vegetative reproduction is much more frequent than sexual reproduction, like it is in present-day protists, and if the mobility of the cells is low. Since the motion of cells or gametes in water is characterized by a Reynolds number (the ratio of the inertial forces to the viscous forces) smaller than one [16], clonally related cells will tend to remain in each others vicinity, and therefore a clonal distribution of cells and gametes is expected, rather than a well-mixed homogeneous population. This implies that mating types will have a smaller chance of finding a suitable mating partner than in a homogeneous population, since they are unable to mate within their clone, while the undifferentiated gamete type has no reduced opportunity for mating, although most matings will be intra-clonal. As shown in a theoretical study by [17] the "mating kinetics" may strongly influence the optimality of a sexual system.

In order to investigate the effects of spatial population structure on the evolution of mating types, we have analysed this process in a cellular automaton model and compared the results it yields to those of the corresponding non-spatial (mean-field) approximation. Such a comparison allows precise consideration of the kinetics of gamete encounters in the model system and emphasizes the role that spatial aspects of the kinetics might have played in mating type evolution. For detailed descriptions of both models see the *Methods *section.

## Results

The specific questions we address with both the mean-field model and the cellular automaton are the following:

a) Are there reasonable parameter values that allow the coexistence of the mating types and the pan-sexual type?

b) Under what (if any) circumstances is it possible that the mating types exclude the pan-sexual type?

c) Does spatial structure play an important role in the outcome of the mating type competition system?

### Coexistence of the two Mating Types and the Pan-Sexual Type

**Figure 1:**
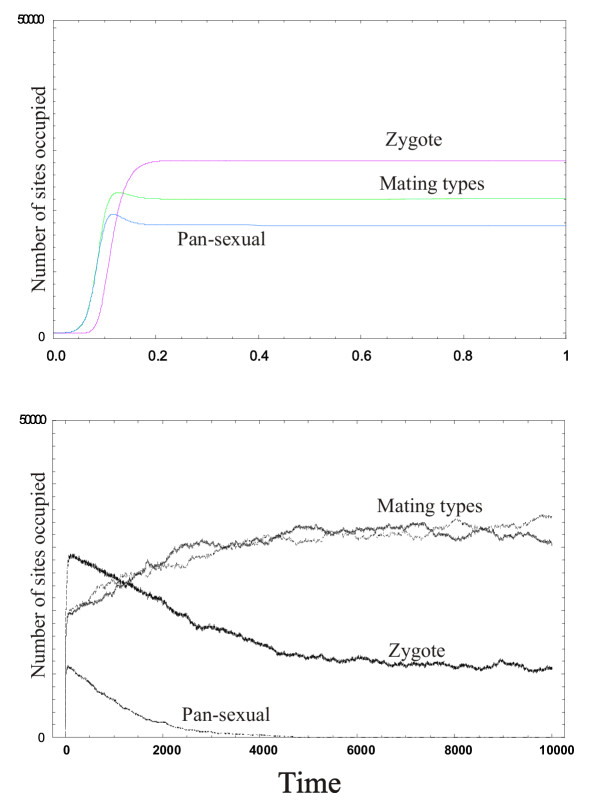
Mating type, pan-sexual and zygote abundances in time, at zero fitness erosion rate (*φ *= 0)) and zero inbreeding effect (*ξ *= 0). Other parameters (in all simulations): Mean-field (upper-panel): birth rate of pre-zygote cells: 0.001; birth rate of post-zygote cells: 0.0015; death rate of pre-zygote cells: 0.12; death rate of post-zygote cells: 0.08; sex rate: 0.0003; germination rate: 15.0; grid size: 90.000. Cellular automaton (lower-panel): birth probability of pre-zygote cells: 0.8; birth probability of post-zygote cells: 0.9; death probability of pre-zygote cells: 0.3; death probability of post-zygote cells: 0.2; sex probability: 0.8; germination probability: 0.8; grid size: 300 × 300 (= 90.000) See the *Methods *section for details.

Numerical solutions to the mean-field model and simulations with the cellular automaton reveal that the system admits a single stable equilibrium state both in the non-spatial and in the spatial setting (see eg. Fig. 1). The actual equilibrium densities depend on the parameters, i.e., on the vegetative growth rates *r*, *R*, *R'*, the vegetative death rates *d*, *D*, *D'*, the germination rate *g*, the sex rate *σ *and the finess erosion rate *φ *in the mean-field, and the corresponding probability parameters in the cellular automaton model. Having explored a broad range of the parameter space – with straightforward constraints on the fitness parameters (birth and death rates), i.e., with *D *≤ *D' *≤ *d *<*r *≤ *R' *≤ *R *– we found that it is the strength of the inbreeding effect (the difference of *D *and *D' *and that of *R' *and *R*) and the rate of fitness erosion *φ *that has the most interesting effects on coexistence. Changing the remaining parameters – the sex rate and the germination rate – within reasonable limits (*σ *> 0, *g *> 0) does not affect the results in a qualitative sense.

We have scaled the inbreeding effect into a single parameter *ξ*, defined by the equations



**Figure 2:**
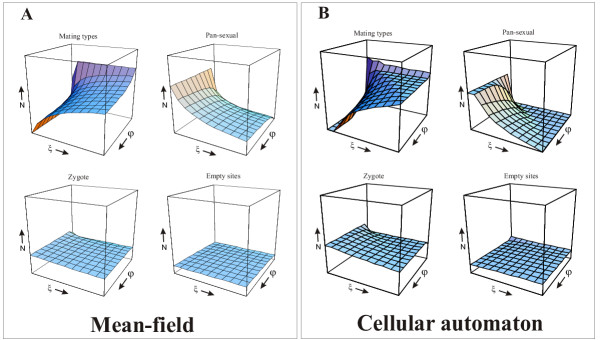
Simulation results: A) mean-field: fitness erosion rate range *φ *: 0.0 → 20.0; inbreeding effect range *ξ *: 0.0 → 1.0; abundance range *N *: 0 → 90.000. B) cellular automaton: fitness erosion probability range *φ *: 0.0 → 1.0; inbreeding effect range *ξ *: 0.0 → 1.0; abundance range *N *: 0 → 90.000

**Figure 3:**
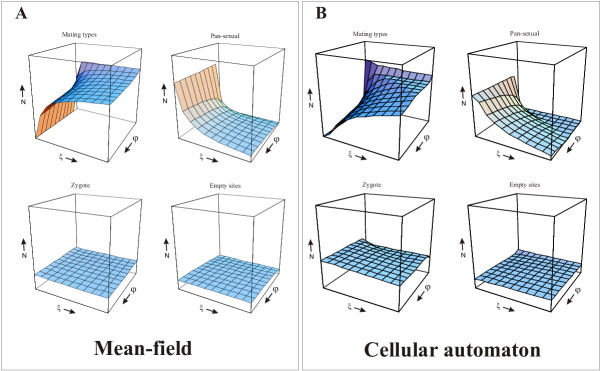
Simulation results with 40% sex rate (sex probability) reduction in the pan-sexual strain. Scales as in Fig. 2. A) mean-field B) cellular automaton

*D' *and* R' *have been replaced by *D*_*ξ *_and *R*_*ξ *_both in the mean-field model and in the spatial simulations, with *ξ *changing from 0 to 1 along the "inbreeding effect" axis of the graphs in Fig. 2 and Fig. 3. *ξ *= 0 represents no inbreeding effect (i.e., the vegetative cells germinated from outbred zygotes have the same fitness as those produced by inbred zygotes), and *ξ *> 0 means a fitness difference in favour of outbred offspring.

Fig. 2 shows the equilibrium densities of the mating types and the pan-sexual type, the zygotes and the empty cells across a range of the *ξ *– *φ *projection of the parameter space, for both the mean-field model (Fig. 2a) and the cellular automaton (Fig. 2b). It is obvious from the graphs that the sum of mating types, pan-sexual and zygote equilibrium densities (and thus the equilibrium density of empty sites) is almost unaffected by the focal parameters, but the relative frequencies of the mating types and the pan-sexual type vary across the *ξ *– *φ *plane. This applies to both the mean-field and the spatial model.

### Role of space

Fig. 2a and Fig. 2b might look quite alike at first sight, suggesting that spatial constraints like short-range interactions and limited dispersion might not play a decisive role in the dynamics of the gamete type competition system. Upon closer inspection of the data, however, this impression turns out to be wrong. Even though the general shapes of the 3D graphs are similar for the non-spatial and the spatial model, there are important differences between them affecting mainly the persistence of the pan-sexual population.

One of these differences shows up in the biologically significant case of very small *ξ *and *φ *values. In the mean-field model, at *ξ *= 0, that is, at no fitness advantage for outbreeding, the pan-sexual strain excludes the mating types for any positive rate of fitness erosion (*φ *> 0). At *φ *= 0 (no fitness loss during vegetative multiplications), on the other hand, it is the mating types who win for any *ξ *> 0. At *ξ *= 0 = *φ*, the mating types and the pan-sexual type coexist, and the same applies to any parameter combination satisfying *ξ *≠ 0 ≠ *φ*. Thus we can say that the non-spatial (mean-field) model allows coexistence for almost any parameter combination, except for the biologically less feasible margins of the parameter plane. It predicts in general that both the mating types and the pan-sexual type should have persisted, even if at variable relative frequencies. The cellular automaton model yields a different prediction, admitting the exclusion of the pan-sexual type, i.e., the victory of the two mating types on a considerable section of the parameter plane, including the *ξ *= 0 = *φ *point and its close (and biologically the most realistic) neighbourhood (cf. Fig. 1).

### Alternative adaptations?

One might guess that in the spatial model the ultimate exclusion of the pan-sexual strain – wherever it happens – is the result of its producing too many dormant zygotes. This would mean that the pan-sexual cells are too frequently induced to become sexually competent and that the resulting high mating frequency impairs their ecological competitiveness. With this hypothesis, a logical next question to ask is: can the pan-sexual strain prevent its elimination by lowering its sensitivity to the induction of sexual competence? With modified versions of both the mean-field model and the cellular automaton we have simulated the effect of such an "adaptation" (Fig. 3). The only modification made to the original models was the reduction by 40 percent of the chance that a pan-sexual cell gets induced by a neighbouring gamete resulting in mating. As it is obvious from a comparison of Figs. 2 and 3, this does not solve the problem of the pan-sexual strain – to the contrary, the chances of the mating types to displace the pan-sexual are even slightly better in the modified models for the largest part of the parameter space. In the mean-field model the relative frequency of the pan-sexual population at equilibrium is smaller almost everywhere except for small nonzero values of the inbreeding effect (compare Figs. 2a and 3a). In the cellular automaton the pan-sexual strain does a little better for very high values of both the inbreeding effect and the fitness erosion rate, but suffers more everywhere else compared to the original model without sex rate reduction (compare Figs. 2b and 3b).

## Discussion

There are a few conclusions that apply to any simulation regardless of its being non-spatial or spatial. Not surprisingly, increasing the fitness advantage of outbreeding *ξ *favours the mating types, because all their sexual interactions produce outbred offspring, while part of the matings of pan-sexual gametes always produces inbred offspring with a smaller fitness. Less obviously, increasing the fitness erosion rate *φ *benefits the pan-sexual type in general, because its effective sex rate is higher: every mating attempt of a pan-sexual gamete can be successful, unlike for the mating types which refuse inbreeding. Therefore the pan-sexual type has more chance than the mating types to reset its eroded fitness to the post-zygote level through mating. The faster the fitness erosion, the more pronounced the advantage of being pan-sexual, hence the more frequent the pan-sexual strain becomes.

In the mean-field model the coexistence of mating types and the pan-sexual type at *ξ *= 0 = *φ *is a spatially unrobust phenomenon. It is highly dependent on the assumption that the system is well-mixed, i.e., each cell encounters other cells of each type with a probability exactly proportional to the relative frequency of that particular type within the whole habitat. It is the breaking of this interaction symmetry in the cellular automaton that gives the mating types a definite advantage compared to the pan-sexuals, even at *ξ *= 0 = *φ *(see Fig. 1). The detailed mechanism is as follows: At *ξ *= 0 it makes no difference whether the mating is inbred or outbred, and at *φ *= 0 the fitness advantage once obtained in a single event of sex cannot be lost. Since dormant zygotes do not die, empty sites can only be produced by the death of vegetative cells, but the death rates are all equal and independent of gamete type, because (after a short transient period) every vegetative cell is in the post-zygote state. For the same reason the birth rates are also equal for all the vegetative cells, so the only factor that can make a difference between the cell types is the availability of empty sites: the limiting "resource" for reproduction. In the mean-field model the empty sites are equally available to any cell, so the growth rates of the pan-sexual and the mating type strains are identical in the long run, hence their coexistence. In the cellular automaton, however, each strain develops patches. The mating type strains do not have sex at all within their own patch, only at the interface with the patches of other strains. The pan-sexual strain has sex all the time everywhere in the habitat, therefore a larger part of its population is in the dormant zygote state. It is for this reason that at the interface with the mating type patches the pan-sexual strain has a smaller supply of vegetative invaders and thus a smaller chance to capture an empty site there. This results in a travelling front between a mating type patch and a pan-sexual patch and ultimately in the demise of the pan-sexual population altogether. This effect can even overcompensate a small disadvantage for the mating types arising from increasing the rate of fitness erosion *φ *slightly above 0, therefore the close neighbourhood of the *ξ *= 0 = *φ *point on the parameter plane belongs to the mating types as well. We think that it is exactly this mechanism that makes the mating types victorious in the spatial model at many parameter combinations that allowed for coexistence in the mean-field approximation. The elementary events at the interfaces between patches of different gamete types have a profound effect on the ultimate outcome of their competition at the larger spatial scale of the whole habitat.

An alternative explanation for the difference of mean-field and cellular automaton results could be that it is the finite size effect that kills off the pan-sexual population from the spatial model at many parameter combinations. Indeed, the cellular automaton is a finite system, the margins of the state space of which are sinks, but looking at the striking difference of the behaviours of the frequency trajectories at *ξ *= 0 = *φ *for example (or anywhere else where the mating types take over) in the two models proves that it is not stochastic drift but a real dynamical trend that eliminates the pan-sexual strain in the cellular automaton (see Fig. 1). The equilibrium value for the pan-sexual type is so far from zero in the mean-field model and its decrease to zero so steady in the cellular automaton that drift as the cause of the difference can be safely ruled out. Moreover, if the pan-sexual strain could be drifted to extinction, so could the mating types, but in fact we have never obtained ambiguous outcomes: sufficiently long replicate simulations always yield the same result. This applies to the whole range of the parameter space.

In order to explain the net effect of sex rate reduction on the fitness, and thus on the survival chances of the pan-sexual population one has to consider two different aspects. On the one hand, sex rate reduction decreases the relative fitness of the pan-sexual strain, because it decreases the frequency of both its inbred and outbred matings, the means of keeping fitness high. This negative fitness effect is most pronounced at high rates of fitness erosion *φ*. On the other hand, less frequent sex yields fewer zygotes, i.e., fewer dormant cells with 0 growth rate (recall that zygotes do not multiply and do not die). If the populations are viable, i.e., if they have a vegetative growth rate higher than 0, then less frequent mating (dormancy) is beneficial in terms of the average fitness of the pan-sexual population. This effect dominates at low values of *φ*, where the fitness advantage of sex does not vanish too fast. A comparison of Figs. 2 and 3 shows that neither these effects are strong, but both are detectable. The net influence on the mean-field model is quantitative, the size of the parameter domain of coexistence is not much affected. In the cellular automaton model the overall effect of sex rate reduction is a slightly larger domain of coexistence: the pan-sexual strain cannot exclude the mating types at high fitness erosion rates, and it is somewhat more persistent at medium values of *φ*. In all, it is quite obvious that sex rate reduction is not an efficient strategy for the pan-sexual strain to avoid exclusion by the mating types.

There is a logical possibility that asymmetric cell fusion has evolved for other reasons than and prior to sex and has subsequently been incorporated in the evolution of a full sexual cycle (the sequence of syngamy, karyogamy and meiosis). In that case sex would have been asymmetric from the start. This speculative idea has been analysed theoretically by [18] (see also [19]). The present analysis clearly does not apply to that scenario, but implicitly explains why sexual asymmetry did not disappear once evolved.

## Conclusions

Assuming that sexual reproduction confers some average fitness advantage compared to simple clonal multiplication, and also supposing that the more genetically different the fusing gametes are the bigger the fitness benefit of the offspring can be, we show that a population consisting of two mating types can displace a pan-sexual population which is otherwise similar to the mating types in all other respects. In the most realistic domain of its parameter space (i.e., at low rates *φ *of the erosion of sexually gained fitness, and very slight extra fitness benefits for heterothallic – outbred – matings, *ξ*) our spatial (cellular automaton) model shows the evolution towards exclusively two mating types, whereas the non-spatial model of the same system with the same parameters predicts the coexistence of the mating types and the pan-sexuals. Thus, taking for granted that sex is profitable in evolutionary terms, we offer a basically ecological answer to the question why two mating types can be better than just one. This is, however, only a solution to half of the problem of the optimal number of mating types. Could a third, a fourth, a fifth etc. mating type invade the same system? These questions arise on a very general level in relation to the origin of sexual asymmetry, and they call for a more extended theoretical approach in the future.

## Methods

### The Mating Type Competition System

**Figure 4:**
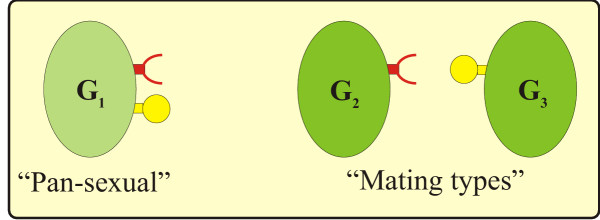
Supposed recognition molecules on the cell surface of the "pan-sexual" type (G1) and the two mating types (G2 and G3)

The basic setup of our model is similar to that of [11]. The model organism is an aquatic unicellular 'alga' with a haplontic life cycle. Three different types of haploid cells compete for space and reproduce both vegetatively and sexually. During the periods between instances of sexual reproduction, the cells multiply vegetatively, producing genetically identical daughter cells. When entering the sexual cycle, a vegetative cell turns into a gamete that can fuse with another gamete. In their gamete stage the three types of cells differ in their mating capacities as represented by different configurations of recognition molecules on the cell surface, as shown on Fig. 4.

The first gamete type G_1 _is 'pan-sexual' and can mate with any potential partner including its own type, while the other two, G_2 _and G_3_, are mating types, unable to mate with their own kind. Thus the system allows four kinds of matings: G_1_.G_1_, G_1_.G_2_, G_1_.G_3 _and G_2_.G_3 _of which only the last one involves both mating types.

In this basic model we furthermore specify the following assumptions. The fitness of a vegetatively produced daughter cell is equal to (or lower than, see below) that of its parent. Sexual fusion produces a dormant zygote which upon germination gives rise to haploid vegetative cells through meiotic division, in which the parental gamete types segregate as if determined by a mendelian pair of alleles. To these meiotic products – "post-zygote" vegetative cells -a higher fitness, i.e., a higher division rate and/or a lower death rate, is attributed than to "pre-zygote" vegetative cells not having gone through a sexual cycle in the near past. That is, we assume that sexual offspring have an immediate short-term fitness advantage over asexually derived daughter cells. The actual advantage may be dependent on whether the zygote has been produced by "outbreeding" (with at least one of the gametes involved belonging to one of the two mating types) or "inbreeding" (both gametes pan-sexual). In general we may, but need not, assume that inbred zygotes yield vegetative cells of somewhat less (but still positive) fitness advantage than outbred zygotes. Note that here "outbreeding" and "inbreeding" mean mating between different and identical gamete types, respectively, i.e., we assume – without specifying the precise nature of this outbreeding advantage – that mating between different gamete types may result in fitter offspring on average than mating between cells of the same (pan-sexual) gamete type. The simplest possible genetic mechanism with this effect might be the production of recombinant offspring carrying fewer (slightly) deleterious alleles than both parental genotypes. This mechanism will be operative more often in heterotypic than in homotypic matings, because among the latter a larger proportion will involve selfing (mating between genetically (almost) identical genotypes). The fitness advantage of sexually derived vegetative cells fades away in time during successive rounds of vegetative reproduction (fitness erosion due to the accumulation of harmful mutations), but it can be re-gained through another sexual event. This means that post-zygote cells return to the pre-zygote state when they are not involved in a new sexual cycle for a sufficiently long time.

As for the ecology of the system, we assume that the habitat consists of a limited amount of sites that cells can occupy, and that the three cell types are competing for these sites. Death events leave empty sites behind, which can be occupied later by new offspring. The chance of a newborn cell to settle is proportional to its division rate and the number of empty sites available. In accordance with what has been said earlier about the fitness advantages of sex, three different division rates and death rates are possible: one for pre-zygote, the second for inbred post-zygote, and the third for outbred post-zygote vegetative cells. The straightforward fitness order of these three types is: *W*_*pre*-*zygote *_<*W*_*post*-*zygote*,*inbred *_≤ *W*_*post*-*zygote*,*outbred*_.

The fusion of two gametes produces a zygote of double size compared to a gamete, and the zygote enters a dormant state with zero rates of division and death. Zygotes leave dormancy at a constant rate, giving rise to post-zygote vegetative cells which inherit the mating type of the gametes they are produced by, and gain fitness according to whether the mating was of the inbreeding or the outbreeding type.

**Figure 5:**
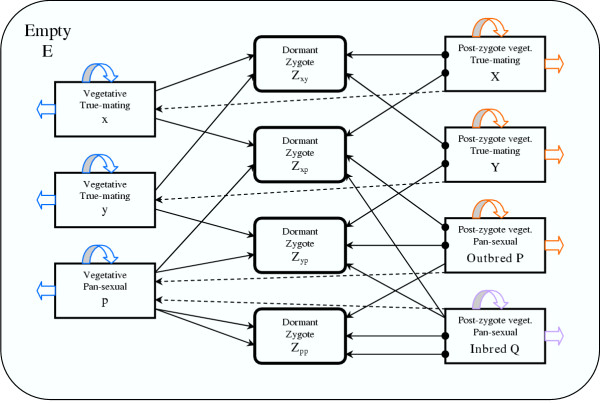
Box diagram of the mean-field model. Box arrows: death of vegetative cells; loop arrows: clonal division; full arrows: sexual fusion; dot-headed arrows: germination; dashed arrows: fitness erosion

Fig. 5 is a diagram of the possible state transitions in the mating type system. The number of possible states for a site is 12 (including the empty state), according to the type of the cell occupying the site. Thus a site can be in any one of the 3 types of pre-zygote vegetative, 4 types of different zygote, 4 types of post-zygote vegetative, and the empty state.

### The Nonspatial Model

Based on Fig. 5, the mathematical formulation of the nonspatial (mean-field) model for the competitive mating type system is straightforward; the differential equations for the 12 site-states are:



where *x*, *y *and *p *are the numbers of sites occupied by pre-zygote vegetative cells (*x *and *y*: mating types, *p*: pan-sexual type), *Z*_*xy*_, *Z*_*xp*_, *Z*_*yp *_are the sites of outbred, and *Z*_*pp *_are those of inbred zygotes. Similarly, *X*, *Y *and *P *are sites of outbred, *Q *are those of inbred post-zygote vegetative cells. *E *is the number of empty sites within the habitat. The parameters of the model are listed and described in Table 1.

The right-hand side of the differential equations for the sites occupied by pre-zygote vegetative cells (*x*, *y *and *p*) has three terms. The first defines the vegetative fitness of the corresponding cell type (divisions and deaths under the competitive effect of all cell types present in the habitat), the second is the outflow from the pre-zygote vegetative state due to sex, and the third is the inflow due to the fitness erosion of post-zygote vegetative cells. Zygotes have no vegetative fitness; the first term in their differential equations is the inflow due to sex, the second is the outflow due to germination. Post-zygote vegetative cells have a vegetative fitness different from that of pre-zygotes (first term); they form zygotes fusing (after induction to sexual competence) with both pre- and post-zygote cells matching in mating type (second term); their fitness advantage erodes at a constant rate resulting in an outflow into the pre-zygote state (third term), and the germination of dormant zygotes maintains an inflow from the zygote states (fourth term). The number of empty sites is increased by the deaths of vegetative cells (first three terms) and decreased by the number of sites taken by newborn vegetative offspring (fourth term). The total number of sites does not change in time, so the 12 time derivatives sum up to zero.

**Table 1:** Parameters of the non-spatial model:

*r*	pre-zygote birth rate
*R*	post-zygote birth rate (outbred)
*R'*	post-zygote birth rate (inbred)
*d*	pre-zygote death rate
*D*	post-zygote death rate (outbred)
*D'*	post-zygote death rate (inbred)
*σ*	sex rate
*g*	germination rate
*φ*	erosion rate of post-zygote fitness advantage

Analytical solutions to this nonlinear model are out of question. We have chosen to find equilibria via numerical solutions, in order to be able to compare the results to those of the spatial model (see below). In all numerical calculations the initial populations were 10 pre-zygote vegetative cells of both mating types and the pan-sexual type, all other states had 0 initial abundances.

### The Spatial Simulation Model

With assumptions as similar to the nonspatial system as possible, we have implemented a site-based (cf. [20]), spatially explicit stochastic cellular automaton model to which the nonspatial system above is a mean-field approximation. The arena of the spatial model is a set of sites arranged in a 300 × 300 square grid of toroidal topology to avoid edge effects. Each site can be occupied by any one of the 11 cell types (3 pre-zygote, 4 post-zygote vegetative types and 4 types of zygote) or it can be empty. Zygotes occupy two adjacent sites.

**Figure 6:**
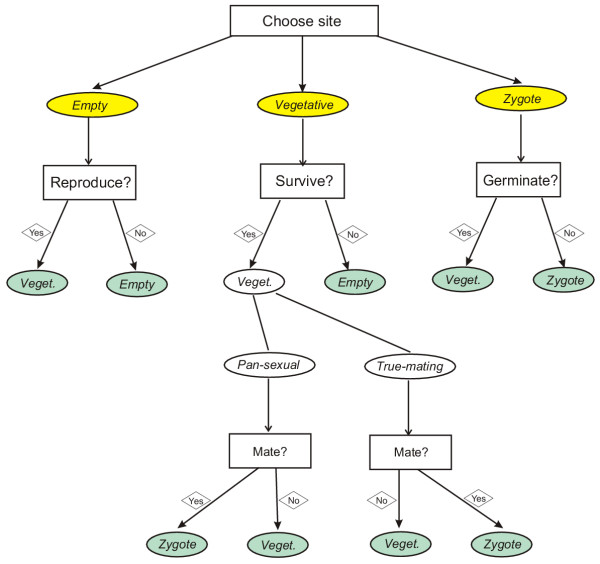
Flow chart of a single site update of the cellular automaton algorithm

The pattern is updated one randomly chosen site at a time, i.e., we use an asynchronous random updating algorithm. Any site chosen for update can be empty, occupied by a vegetative cell, or occupied by a zygote. We specify the algorithm for each of these cases in turn. A schematic diagram of a single step of updating is given in Fig. 6.

#### Empty site update

After updating, an empty site can be occupied by one (and only one) of the vegetatively produced offspring of the cells in the 8 neighbouring sites (i.e., the Moore neighbourhood of the focal site), or it remains empty. Each vegetative neighbour *i *has a chance *p*_*i *_to put a daughter cell into the empty site. *p*_*i *_depends on the vegetative reproduction parameter *β*_*I *_(0 ≤ *β*_*I *_≤ 1)of neighbour *i*. *β*_*I *_is the spatial analogon of *r*_*i *_in the mean-field model, and it takes one of three possible values depending on whether *i *is in the pre-zygote, the inbred or the outbred post-zygote state.

Specifically, the chance of the empty site to remain empty is



so the probability that the offspring of neighbour *i *takes the site is



The rationale behind this formalism is that each neighbour attempts putting an offspring into the empty site with a probability *β*_*i*_, but only one of the candidate offspring survives. The chance of survival is proportional to the reproduction parameter of the mother cell.

#### Vegetative site update

Updating a site occupied by a vegetative cell may result in four possible outcomes: turn the site into the empty state (death), leave it as it was (survival maintaining fitness), change the vegetative status of the resident cell from post-zygote to pre-zygote (survival with fitness erosion), or produce a zygote (sex). The probability of a death event depends on the death probability *δ *of the cell occupying the site, which in turn depends on its vegetative status (pre-zygote, inbred or outbred post-zygote). With a mating partner in one of the neighbouring sites, a surviving vegetative cell may enter the sexual cycle with probability *s *turning itself and a randomly chosen, suitable neighbour into gametes, and mate. The result is a dormant zygote occupying the two neighbouring sites of the fused gametes. A survivor skipping sex may keep its original fitness, or – if it was a post-zygote cell – it can lose its fitness advantage with a probability *f *(which is the spatial analogon of the fitness erosion rate *φ *in the mean-field model).

#### Zygote site update

A zygote can do two things: remain dormant (with probability 1 – *γ*) or germinate (with probability *γ*). A germinated zygote yields two vegetative cells, the mating types of which are the same as those of the gametes which produced the zygote. The vegetative status of the cells thus obtained is post-zygote, and they can be either inbred or outbred, depending on the parental gamete type combination. The daughter cells are positioned at random into the two sites the zygote had occupied.

At time 0 we have populated 2% of the sites by pre-zygote vegetative individuals of both mating types and the pan-sexual type, assigning individuals to sites at random. All other sites were empty at time 0. The simulations were run for 10.000 generations.

## Authors' contributions

RH organized and coordinated the project, collected most of the literature and drafted parts of the manuscript. TC designed and built the models, drafted parts of the manuscript and the figures.
